# Surgical Risk Factors for Delayed Oral Feeding Autonomy in Patients with Left-Sided Congenital Diaphragmatic Hernia

**DOI:** 10.3390/jcm12062415

**Published:** 2023-03-21

**Authors:** Mélina Bourezma, Sébastien Mur, Laurent Storme, Emeline Cailliau, Pascal Vaast, Rony Sfeir, Arthur Lauriot Dit Prevost, Estelle Aubry, Kévin Le Duc, Dyuti Sharma

**Affiliations:** 1 CHU Lille, Clinic of Pediatric Surgery, Jeanne de Flandre Hospital, FR-59000 Lille, France; 2 CHU Lille, Clinic of Neonatology, Jeanne de Flandre Hospital, FR-59000 Lille, France; 3 Center for Rare Disease Congenital Diaphragmatic Hernia, CHU Lille, Jeanne de Flandre Hospital, FR-59000 Lille, France; 4 ULR 2694-METRICS: Medical Practices and Health Technology Evaluation, CHU Lille, Université de Lille, FR-59000 Lille, France; 5 Biostatistics Department, CHU Lille, FR-59000 Lille, France; 6 CHU Lille, Clinic of Obstetrics and Gynaecology, Jeanne de Flandre Hospital, FR-59000 Lille, France

**Keywords:** congenital diaphragmatic hernia, failure to thrive, oral feeding autonomy, gastrostomy, surgical reintervention

## Abstract

Background: Congenital diaphragmatic hernia (CDH) is a rare disease associated with major nutritional and digestive morbidities. Oral feeding autonomy remains a major issue for the care and management of these patients. The aim of this study was to specify the perinatal risk factors of delayed oral feeding autonomy in patients treated for CDH. Methods: This monocentric cohort study included 138 patients with CDH. Eighty-four patients were analyzed after the exclusion of 54 patients (11 with delayed postnatal diagnosis, 5 with chromosomal anomaly, 9 with genetic syndrom, 13 with right-sided CDH, and 16 who died before discharge and before oral feeding autonomy was acquired). They were divided into two groups: oral feeding autonomy at initial hospital discharge (group 1, *n* = 51) and nutritional support at discharge (group 2, *n* = 33). Antenatal, postnatal, and perisurgical data were analyzed from birth until first hospital discharge. To remove biased or redundant factors related to CDH severity, statistical analysis was adjusted according to the need for a patch repair. Results: After analysis and adjustment, delayed oral feeding autonomy was not related to observed/expected lung-to-head ratio (LHR o/e), intrathoracic liver and/or stomach position, or operative duration. After adjustment, prophylactic gastrostomy (OR adjusted: 16.3, IC 95%: 3.6–74.4) and surgical reoperation (OR adjusted: 5.1, IC 95% 1.1–23.7) remained significantly associated with delayed oral feeding autonomy. Conclusions: Delayed oral feeding autonomy occurred in more than one third of patients with CDH. Both prophylactic gastrostomy and surgical reoperation represent significant risk factors. Bowel obstruction might also impact oral feeding autonomy. Prophylactic gastrostomy seems to be a false “good idea” to prevent failure to thrive. This procedure should be indicated case per case. Bowel obstruction and all surgical reoperations represent decisive events that could impact oral feeding autonomy.

## 1. Introduction

Congenital diaphragmatic hernia (CDH) is generally associated with ascension of the abdominal viscera towards the thorax, and abnormalities in pulmonary development responsible for pulmonary hypoplasia and structural and functional abnormalities in pulmonary circulation. Its functional consequences are particularly heterogeneous, since some infants are asymptomatic at birth, while others present major failure of the cardio-respiratory adaptation to extrauterine life. The survival rate of infants with CDH has increased during recent decades, but the mortality rate remains at 20% to 30% in tertiary care centers [1]. With the improvement of survival, long-term morbidity of these children has unfortunately increased, cardiovascular and pulmonary (persistent pulmonary hypertension, chronic lung disease, recurrent respiratory tract infections), neurodevelopmental, orthopedic (chest wall and spinal deformities) surgical (hernia recurrence, bowel obstruction), and gastrointestinal complications (gastroesophageal reflux, oral aversion, failure to thrive) associated with CDH have become more visible.

Morbidity of CDH at the age of one year is mainly determined by gastrointestinal and respiratory problems [2].

Children acquire oral feeding autonomy when their oral intake is sufficient to meet nutritional and caloric requirements, without any nutritional support (except caloric fortification) to permit adequate growth. Moreover, the occurrence of failure to thrive in CDH survivors is likely multifactorial caused by catabolic stress in the neonatal period, suboptimal nutritional intake due to gastroesophageal reflux disorder and/or oral aversion, and persistent chronic lung disease with an increased caloric requirement [3,4,5]. About one third of CDH survivors have significant failure to thrive, requiring enteral nutrition by a gastrostomy in the first year of life [6]. Oral aversion has an estimated incidence rate of 25% in patients with CDH [5,6] and is one of the most determinant factors that may lead to the need for nutritional support.

Delayed oral feeding autonomy may lead to the need for secondary surgical placing of a gastrostomy, which carries important complication risks and has important practical, psychological, and negative social consequences for parents [7,8].

Many children in this population experience postoperative complications such as bowel obstruction, hernia recurrence, and surgical reintervention [9,10,11,12]. We postulated that some surgical repair conditions and surgical complications may delay oral feeding autonomy; therefore, we decided to focus in the present study on these modifiable factors. Thus, the aim of this study was to identify antenatal and neonatal factors, especially surgical events associated with delayed oral feeding autonomy in patients with CDH.

## 2. Materials and Methods

### 2.1. Population

#### 2.1.1. Inclusion Criteria

This monocentric retrospective study reviewed all neonates admitted for the management of CDH at the Neonatal Intensive Care Unit (NICU) of Lille University Hospital from January 2009 to December 2018.

In the Nord-Pas-de-Calais region of France (4.5 million inhabitants; 55,000 births/year), all infants with a CDH diagnosis were referred to the Lille University Hospital and were enrolled in a prospective follow-up study.

#### 2.1.2. Exclusion Criteria

To form a uniform cohort, the following were excluded from the study:

- Infants with a chromosomal anomaly or a genetic syndrome;
- Delayed diagnosis of postnatal CDH (>24 h after birth);
- Right-sided or bilateral defect;
- Morgagni hernia;
- Death occurring before discharge and before oral feeding autonomy (OFA) was acquired.

### 2.2. Treatment and Surgical Management

Management of infants with CDH was based on the EURO Consortium guidelines [13] and national French guidelines [14] taking into account that Storme et al. showed that implementing a nationwide protocol for CDH was a key element in reducing mortality and morbidity [15].

Surgical repair was usually carried out after a short period of cardiorespiratory stabilization. All patients with antenatal diagnosis of CDH were operated on through subcostal laparotomy. Thoracoscopic repair was proposed to patients with post-natal diagnosis of left-sided CDH, who did not have respiratory distress and without hemodynamic impact. When primary repair was not possible, a Gore-Tex^®^ (Flagstaff, AZ, USA) patch closure was performed.

In our center, gastrostomy tube placement during the initial surgery was usually decided by the surgeon; depending essentially on the defect size, gastrostomy was placed for C or D defect from the Congenital Diaphragmatic Hernia Study Group classification. The defect of anterior diaphragmatic pillar and intrathoracic position of stomach also had an influence on gastrostomy placement.

Fundoplication was never performed during the initial surgery.

We systematically placed a chest tube with gentle suction at the end of the surgery to control pleural pressure and treat postoperative pleural effusion or chylothorax.

Sodium hyaluronate-carboxycellulose membrane (Seprafilm^®^; Deerfield, IL, USA) to prevent postsurgical adhesions was not applied during the study period.

Intrathoracic drainage was systematically left in all patients regardless of the approach with an aspiration pressure of −5 cm H_2_O, corresponding to the physiological intrapleural pressure.

Extracorporeal membrane oxygenation (ECMO) support was always veno-arterial.

### 2.3. Data Collection

Data were collected retrospectively by a manual search of individual medical records and operative reports. All postnatal data concerned events that occurred during the neonatal period from birth to the first hospital discharge (except OFA that can occur later): postoperative pleural effusion, hernia recurrence, bowel obstruction, and surgical reintervention.

### 2.4. Method

The primary endpoint was delayed OFA. OFA was considered as acquired when all nutritional needs were ingested, without any nutritional support except caloric enrichment, for a gain of weight in the standards on the INSERM growth curve [16].

**Figure 1:**
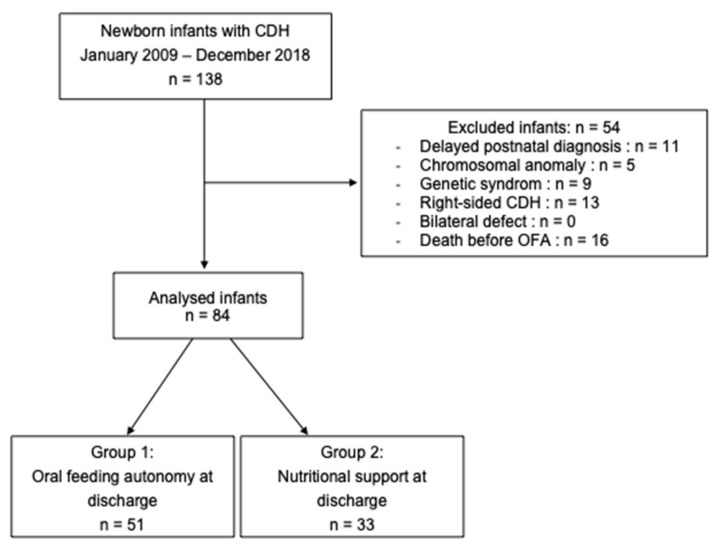
Flowchart. Congenital Diaphragmatic Hernia (CDH); Oral Feeding Autonomy (OFA).

Eighty-four patients were analyzed after the exclusion of 54 patients as represented in the flow chart (Figure 1): 11 with delayed postnatal diagnosis, 5 with chromosomal anomaly, 9 with genetic syndrom, 13 with right-sided CDH, and 16 who died before discharge and before OFA was acquired.

To analyze potential risk factors of delayed OFA, we decided to compare antenatal, surgical, and postoperative data in two groups: patients with OFA at discharge (group 1, *n* = 51) and patients requiring nutritional support at discharge (group 2, *n* = 33). For both groups, we recorded demographic and CDH-specific data as follows.

#### 2.4.1. Demographic Data

Demographic variables included gender, gestational age at birth, and birth weight.

#### 2.4.2. Hospitalization Data

We recorded the age at neonatal intensive care unit and hospital discharge.

#### 2.4.3. Antenatal Data

For each patient, we defined the mean o/e LHR, calculated from all available measurements of o/e LHR by antenatal ultrasound.

#### 2.4.4. Surgical Data

We recorded ECMO support placement and data about the initial surgery: surgical approach, type of repair (primary repair or patch of Goretex^®^), presence of liver and/or stomach herniation, gastrostomy placement, and operation time.

#### 2.4.5. Postoperative Data

We recorded postoperative pleural effusion, hernia recurrence, bowel obstruction syndrome (defined by feeding intolerance, abdominal distension, vomiting and a suggestive abdominal X-ray), and the need for surgical reintervention (for hernia recurrence, bowel obstruction, abdominal compartment syndrome, or another cause) occurring before hospital discharge.

### 2.5. Adjustment on Patch Repair

In our study, adjustment of the results with patch repair was decided for the following reasons:

- The impact of CDH severity on short- and long-term outcomes (and especially nutritional outcomes) is already well known [6,17,18] and that suggested a need to evaluate the factors potentially associated with our outcome, independent of well-known severity factors of CDH;
- The need for a patch repair is one of the two main neonatal factors, with the requirement of ECMO almost constantly reported as associated with a failure to thrive and/or the need for nutritional support [19,20,21,22]. Because of a very small number of patients requiring ECMO in our population (*n* = 5), patch repair was the best adjustment variable in our study;
- Patch repair is a well-known predictive factor of CDH severity [23,24,25], and incidence of surgical complications (such as bowel obstruction, hernia recurrence, and subsequent abdominal operations) is higher in patients with a patch repair [9,12].

### 2.6. Statistical Analysis

Categorical variables are expressed in terms of frequency and percentage. Quantitative variables are expressed as means ± standard deviation in the case of normal distribution or medians (interquartile range, IQR) otherwise. Normality of distributions was checked graphically and using the Shapiro–Wilk test.

Risk factors for delayed OFA were identified using a logistic regression model, with and without adjusting for the patch repair. Odds ratios and their 95% confidence intervals were derived from models as effect size. No statistical comparisons were carried out for categorical variables with frequency <5.

A *p*-value of 0.05 was used for all significance tests. All analyses were performed using SAS 9.4 (SAS institute, Cary, NC, USA).

### 2.7. Ethical Agreement

Parents of infants were informed of, and agreed to, the collection and use of their infants’ data. The study was approved by the Commission Nationale de l’informatique et des Libertés (CNIL) No. Dec 19-328.

## 3. Results

### 3.1. Population Characteristics

Demographic, hospitalization, antenatal, surgical, and postoperative characteristics of the population are represented in Table 1 and Table 2.

The majority of repairs were performed by subcostal laparotomy (approximately 95.2% of the population). The difference in surgical approach was not calculable because of the small number of events in each group (all patients who had thoracoscopy were in group 1).

Twenty-three (27.4%) patients had a gastrostomy placement during the initial surgery; almost 87% of them had a delayed OFA. Every gastrostomy exposed in this study was primary gastrostomy, placed during initial surgery.

ECMO support was required for 6 patients; one on those patients was excluded because of refractory hypoxemia leading to death at 8 days of life. Consequently, 5 patients (6.0%) analyzed in the study had ECMO; the difference between the two groups was not calculable. In all cases, VA ECMO was used.

In the cohort, chylothorax was present for two patients in the study; both of them were in group 1. 

We collected postoperative data occurring before the first hospital discharge. Three patients (3.6%) had a diaphragmatic hernia recurrence (*n* = 2 in group 1 and *n* = 1 in group 2, *p* non available), and they all had a redo surgery before the first hospital discharge. In addition, bowel obstruction was reported in 11 patients. Of these patients, five needed surgical intervention for adhesions (all of them were in group 2) while the other patients were treated medically.

Three patients needed a second surgery for other causes: 1 patient for abdominal compartment syndrome, 1 patient had a change in gastrostomy site associated with a patch size reduction, and 1 patient had a pneumperitoneum caused by a gastric perforation near the gastrostomy site.

In summary, 11 (13.3%) patients required surgical reintervention before discharge, and almost 73% of them had delayed OFA. Almost half of reinterventions were related to bowel obstruction and 27% to a hernia recurrence.

### 3.2. Factors Associated with Delayed Oral Feeding Autonomy

Adjustment was made for the patch repair. The non adjusted and adjusted odds ratios (OR) for antenatal, surgical, and postoperative data are presented in Table 2.

After this adjustment, gastrostomy placement during the initial diaphragmatic surgery (ORadjusted 16.3 (3.5–74.4); *p* < 0.001) and need for a second surgery whatever the cause (ORadjusted 5.1 (1.1–23.7); *p* = 0.037) remained significantly associated with delayed OFA. Bowel obstruction tended to impact the delay of OFA but without significant effect (ORadjusted 3.7 (0.9–15.7); *p* = 0.078) (Table 2).

## 4. Discussion

The aim of this study was to identify perinatal factors (especially surgical and postoperative events) associated with delayed OFA in patients with left-sided CDH. Our results showed that, after adjustment for patch repair, two main factors remained significantly associated with delayed OFA: gastrostomy placement during initial surgery and surgical reintervention during initial hospitalization.

After adjustment, no significant difference was found between the two groups concerning antenatal (LHR o/e) and “anatomic” (liver and stomach position) data, suggesting that this adjustment ensures better comparability of the two groups. To our knowledge, gastrostomy placement during initial surgery (“prophylactic gastrostomy”) and postoperative events during the neonatal period in this population have never been evaluated as potential predictors of delayed OFA and consequently of the need for nutritional support.

Su et al. [26] studied predictive factors of gastroesophageal reflux in the neonatal period and found that patch repair and ECMO were high risk factors of severe gastroesophageal reflux with fundoplication. Experience of prophylactic antireflux at initial surgery was described in a few studies [27,28,29,30,31,32], but actually none of them has shown persistent beneficial effects on gastroesophageal reflux in the short and long term. For these reasons, fundoplication was never performed during the initial surgery in our center. To prevent nutritional morbidity, surgeons performed prophylactic gastrostomy in the initial surgical repair in CDH patients for these anatomic particularities: C or D defect, intrathoracic stomach, and/or diaphragmatic anterior pillar defect.

Our results indicate that prophylactic gastrostomy is associated with delayed oral autonomy. Indications of fundoplication and gastrostomy tube placement at the surgical repair are not consensual between centers [30]. Prieto et al. recently identified factors associated with gastrostomy or jejunostomy in neonates with CDH during their initial hospitalization, and established a scoring system based on these factors to guide clinical decisions [18]. Even so, there is still no consensus for the timing of the gastrostomy placement. A significant decrease of gastric emptying has been reported with the placement of a gastrostomy tube. Fifty percent of a child population with a normal preoperative gastric emptying developed delayed gastric emptying after gastrostomy placement [33]. In addition, the author reported that delayed gastric emptying after gastrostomy placement was associated with gastroesophageal reflux, and was found in most patients with feeding intolerance [33]. Indeed, worsening or development of gastroesophageal reflux after gastrostomy placement has been reported in another study [34], suggesting that gastroesophageal reflux may also contribute to feeding intolerance and consequently to a delay in OFA. Finally, some authors are reluctant to perform gastrostomy because it may promote oral aversion [5]. Another hypothesis is that patients with a gastrostomy placed during the initial surgery might be less stimulated orally by physicians and family over the long term, consequently leading to a need for prolonged enteral feeding via gastrostomy. However, it is important to remember that gastrostomy for enteral support prevents, or at least limits, failure to thrive for many patients with CDH. In our study, we found that gastrostomy placement in patients with CDH during the initial surgery could delay OFA.

This study highlights the fact that “prophylactic” gastrostomy placement at surgical repair is required to assess individual benefits (prevention of growth failure, only one general anesthesia for diaphragmatic repair, and gastrostomy placement) but bears this risk of delayed OFA.

Gastroesophageal reflux is a frequent complication in survivors of CDH and increases growth disorders, but fundoplication was never performed during the initial surgery in our center. Indeed, indications seem to be not consensual between centers. Dariel et al. found that prophylactic fundoplication was associated with survival without disordered growth [28], while a recent study showed that preventive fundoplication was associated with higher rates of failure to thrive, tube feeds, and oral aversion [31].

The present study also indicated for the first time that surgical reintervention (and maybe bowel obstruction) in patients with CDH during the neonatal period could delay OFA. Several mechanisms may play a role here. We hypothesize that anesthesia and abdominal surgery alter the complex physiopathology of gastrointestinal motility and have an important role in the postoperative ileus [35]. A second surgical intervention also exacerbates the risk of postoperative adhesions that may lead to abdominal pain, altered intestinal mobility, and bowel obstruction. Oral aversion also plays an important role in OFA acquisition, with an estimated incidence rate of 25% in patients with CDH in several series [5,6]. One of the mechanisms that may be referenced to support our findings is that pharyngeal stimulation by the nasogastric tube, and the pause in oral feeding during the period of bowel obstruction, may contribute to a delay in development of the swallowing reflex and the suckling mechanisms. These mechanisms have also been mentioned to explain why patients with prolonged endotracheal intubation develop oral aversion [6]. In our study, 11 (13.3%) patients required surgical reintervention before discharge. Another study reported a similar incidence of second surgery in the first three months of life [2]. Nobuhara et al. also reported that the most commonly performed second surgery was re-exploration for small-bowel obstruction [3]. Some risk factors have recently been evaluated [36].

### 4.1. Limitations of the Study

It is difficult to compare our rates of complications with other studies, even if our population was comparable with other studies in terms of gestational age and birth weight [9,37]. We only assessed neonatal complications occurring before the first hospital discharge while most other studies have reported a long-term follow up of CDH patients [10,12,38].

We focused our intention on postnatal data generated before the first hospital discharge, while surgical complications (bowel obstruction and/or surgical reintervention or CDH recurrence) may also occur later. It would be interesting to assess if long-term surgical complications may also compromise OFA.

### 4.2. Conclusions

Gastrostomy placement during initial surgery and a surgical reintervention during the neonatal period were significantly associated with delayed OFA in infants with CDH. Bowel obstruction occurrence before the first hospital discharge might also impact OFA acquisition.

Patients with CDH have nutritional and digestive neonatal morbidities. We need to be very careful in matters concerning nutritional needs and oral feeding, especially in children presenting with antenatal predictive factors of CDH severity. Risks and benefits should be assessed individually before placement of a prophylactic gastrostomy during initial surgery.

These data had already had an important impact on our primary gastrostomy placement management. Actually, in our team, gastrostomy is not placed during the initial surgery anymore, even for large defects. By following assiduously the patient nutritional and digestive evolution, placement of a gastrostomy may be discussed and proposed secondly.

## Figures and Tables

**Table 1:** Demographic and hospitalization characteristics of the population.

Characteristics	Total *n* = 84	Group 1 *n* = 51	Group 2 *n* = 33	*p*-Value
**Demographic data**				
Gender-male	45 (53.6)	26 (51)	19 (57.6)	0.55
Premature birth	5 (6)	3 (5.9)	2 (6.1)	N/A
Gestational age	39 (1.8)	39.1 (1.4)	38.8 (2.3)	0.44
Birth weight	3250 (575)	3287 (463)	3193 (463)	0.46
**Hospitalization data**				
Age at NICU discharge (days)	34.5 (16;60)	22 (14;40)	53 (38.90)	<0.001
Age at hospital discharge (days)	38 (20;69)	23 (16;41) ^1^	68 (39;104)	<0.001

*n*, number; NICU, neonatal intensive care unit; o/e, observed/expected. Categorical data are expressed as number (%). Continuous data are expressed as mean (standard deviation) or median (interquartile range, IQR). ^1^ 3 missing values.

**Table 2:** Surgical data and postoperative events of the population.

Characteristic	Total *n* = 84	Group 1 *n* = 51	Group 2 *n* = 33	OR_crude_ (95% CI)	*p*-Value	OR_adjusted_ (95% CI) ^1^	*p*-Value
**Antenatal data**							
LHR	51.5 (16.6)	56.2 (17.0) ^1^	44.7 (13.5) ^2^	1.8 (1.1–2.8) ^3^	0.008	1.1 (0.7–1.9) ^3^	0.58
**Surgical data**							
ECMO support	5 (6.0)	2 (3.9)	3 (9.1)	N/A	N/A	N/A	N/A
Laparotomy	80 (95.2)	47 (92.2)	33 (100.0)	N/A	N/A	N/A	N/A
Thoracoscopy	4 (4.8)	4 (7.8)	0 (0.0)	N/A	N/A	N/A	N/A
Liver up	26 (32.1)	10 (20.4) ^4^	16 (50.0) ^5^	3.9 (1.4–10.4)	0.007	1.5 (0.4–5.2)	0.53
Stomach up	49 (60.5)	24 (49.0) ^6^	25 (78.1) ^7^	3.7 (1.3–10.2)	0.011	2.2 (0.7–6.8)	0.16
Patch repair	27 (32.1)	8 (15.7)	19 (57.6)	7.3 (2.6–20.3)	<0.001	N/A	N/A
Primary gastrostomy	23 (27.4)	3 (5.9)	20 (60.6)	24.6 (6.3–95.9)	<0.001	16.3 (3.5–74.4)	<0.001
Operative time (minutes)	57.5 (40; 65)	50 (40; 60) ^8^	60 (50; 80) ^9^	1.5 (0.9–2.6) ^10^	0.086	1.1 (0.6–2.0) ^10^	0.64
**Postoperative events**							
Postoperative pleural effusion	30 (35.7)	11 (21.6) *	19 (57.6)	4.9 (1.8–12.9)	0.001	2.8 (0.9–8.3)	0.056
Hernia recurrence	3 (3.6)	2 (3.9)	1 (3.0)	N/A	N/A	N/A	N/A
Bowel obstruction	11 (13.3)	4 (7.8)	7 (21.2)	3.2 (0.8–11.9)	0.087	3.7 (0.8–15.7)	0.078
Surgical reintervention	11 (13.3) ^11^	3 (6.0)	8 (24.2)	5.0 (1.2–20.6)	0.025	5.1 (1.1–23.7)	0.037

*n*, number, OR, odd ratio, CI; confidence interval, o/e LHR; observed/expected lung-to-head ratio (%), Categorical data are expressed as number (%), Continuous data are expressed as median (interquartile range, IQR), ^1^ 12 missing values, ^2^ 6 missing values, ^3^ Per 10% decrease, ^4^ 2 missing values, ^5^ 1 missing value, ^6^ 2 missing values, ^7^ 1 missing value, ^8^ 4 missing values, ^9^ 4 missing values, ^10^ Per 25 min increase, ^11^ 1 missing value, * including 2 chylothorax.

## Data Availability

Data available on request due to restrictions eg privacy or ethical.
